# Prevalence, determinants and attitude towards herbal medicine use in the first trimester of pregnancy in Cameroon: A survey in 20 hospitals

**DOI:** 10.1371/journal.pgph.0000726

**Published:** 2022-08-30

**Authors:** Aminkeng Zawuo Leke, Helen Dolk, Maria Loane, Karen Casson, Nkwati Michel Maboh, Susan Etta Maeya, Lerry Dibo, Pauline Bessem Nyenti, Armstrong Obale, Derick Etiendem

**Affiliations:** 1 Centre for Maternal, Fetal and Infant Research, Institute for Nursing and Health Research, Ulster University, Jordanstown, United Kingdom; 2 Centre for Maternal and Infant Research, Health Research Foundation (HRF), Buea, Cameroon; University of California San Francisco, UNITED STATES

## Abstract

To examine the prevalence, determinants and attitude towards herbal medication (HM) use in the first trimester of pregnancy in Cameroon women. Between March to August 2015, we surveyed 795 pregnant women attending 20 randomly selected urban or rural hospitals in South West Cameroon on first trimester orthodox medication (OM) and HM use. Data was obtained by interviews using structured questionnaires. First trimester HM use was reported by 293 (36∙9%) women, 76% of whom used it in combination with OM. The most frequent indication for taking HM was prevention/treatment of anaemia (26∙3%). The HM were usually self-prescribed (33∙3%) or by family (56∙2%), and obtained from the woman’s own garden (69∙3%). Twenty percent of women believed that HM was always safe to take in pregnancy, compared to 69.3% for OM. Intake of HM was significantly influenced by women’s opinion on OM or HM safety—the odds of taking HM was 3 time higher among women who were unsure about the safety of OM (AOR: 3∙0, 95%CI = 1∙5–6∙1), while women who thought HM were never safe or who were unsure about its safety, were 91% or 84% respectively less likely to take HM compared to women who believed HM were always safe. We identified a high prevalence of HM use and concomitant use with OM, strongly influenced by women’s perception of HM and OM safety. These findings indicate the need for WHO to specifically address safety in pregnancy in its policy to integrate traditional medicine use into existing healthcare systems in Africa.

## Introduction

The World Health Organisation (WHO) defines traditional medicine (TM) as “the sum total of the knowledge, skill, and practices based on the theories, beliefs, and experiences indigenous to different cultures, whether explicable or not, used in the maintenance of health as well as in the prevention, diagnosis, improvement or treatment of physical and mental illness” [1]. In African TM, spirituality plays a central role [2, 3]. Herbal medication (HM) remains the predominant element of TM [4].

Over the past two decades TM has been gaining attention in the African continent, following its repression during the colonial period [2, 5]. This is mainly due to failure of orthodox medication (OM) to treat resistant infections such as malaria and emerging conditions such as HIV/AIDS, cancer, diabetes, hypertension and now COVID-19, compounded by a strong cultural belief in the efficacy of HM, high cost of OM, limited access to healthcare facilities [2, 6, 7]. There is also a growing sense of recognition of the fact that the potential of the over 74,000 plant species in Africa remains largely unexploited [2]. The WHO estimates that 80% of the populations in Africa use TM to meet their primary health care needs. For many living in rural areas TM is the only available, accessible, and affordable source of health care [8]. Established since 2000, the WHO has an active plan to integrate TM into national healthcare systems in Africa [9, 10].

Medicine use in pregnancy, whether orthodox or herbal, is a special concern because the risk and benefits of treatment need to be weighed for both mother and baby [11]. Many OM have limited safety data for pregnancy [12], and the situation is even more serious for HM [13]. The first trimester of pregnancy is of particular concern in relation to the teratogenic potential of medicine use [14].

Worldwide, HM is commonly used by pregnant women to alleviate various pregnancy symptoms or complications, including relief from nausea and vomiting, improvement of fetal growth, stimulation of labour and delivery, prevention of premature labour and spontaneous abortion, and aiding placental expulsion [12, 15–18]. The prevalence of HM use in pregnancy varies based on geographic location, ethnicity, cultural traditions, and socioeconomic status [12]. For example, a multinational study reported a prevalence of 27.7%, 11.9%, 51.9%, 26.6%, 17.9% and 43.8% among pregnant women from Western Europe, Northern Europe, Eastern Europe, North America, South America, and Australia respectively [18]. Generally, HM use among pregnant women in African countries is higher compared to other parts of the world, with prevalence of up to 90% reported [4, 15, 19].

Studies conducted in Africa have reported different factors that influence HM use during pregnancy. These include respect of cultural norms, religious affiliation, perceived efficacy and safety of HM over OM, poverty, low level of education, high prevalence of diseases, limited access to mainstream maternity care, limited care facilities in rural settings, and availability and accessibility of traditional birth attendants [4, 15, 20].

While a number of studies have been conducted in Africa on HM use during pregnancy, there is still a paucity of data in many African countries such as Cameroon. In a recent review on HM use during pregnancy, the authors identified 50 studies from only 13 of the 54 countries in Africa, with none from Cameroon [12, 15]. In general, these studies have paid limited attention to major safety factors such as concurrent use of HM and OM, women’s perception of safety, sources of safety advice, or HM use in the first trimester [12, 15]. The limited focus on HM safety issues in pregnancy is also reflected in its absence from the WHO’s strategy to integrate TM into healthcare systems in Africa [9, 10]. There is also limited evidence of how women’s attendance of public or private hospital may influence their use of HM use.

The need to reorganise and integrate traditional medicine practice into the Cameroon national healthcare system has been identified since 1970 [21]. However, traditional medicine practice in Cameroon still remains largely uncoordinated and unregulated and has not yet incorporated the WHO’s existing recommendations [10, 22].

We present in this paper a survey in the Cameroon on the prevalence, determinants and attitude to HM use in the first trimester of pregnancy. Details of OM use by women of this study had been presented in a previous publication [23]. The paper also discusses how the study results, in the context of the literature, can inform the WHO’s strategy for integrating TM into healthcare systems in Africa. This is particularly relevant with the increase in use of traditional medicine during the Ebola virus and COVID-19 outbreaks.

## Materials and methods

### Study design

This study was a cross- sectional hospital-based medication utilisation survey of pregnant women involving the use of questionnaire-based interviews. Women who were between 3 and 7 months pregnant were asked about medicines they had taken during their first trimester (0-3months). The survey was conducted between March to August 2015 (six-month period).

### Population

This study involved pregnant women attending urban and rural hospitals (for antenatal or other care) in the South West Region (SWR) of Cameroon.

### Inclusion and exclusion criteria

Urban and rural hospitals were eligible to participate if they had an annual delivery rate of over 200 and 100 births respectively. All pregnant women attending the selected hospitals on the days the researchers were in attendance (registered for antenatal clinics or not) were eligible to participate. To limit recall bias and to target the period within first trimester, only women with a gestation of three to seven months were eligible to participate in the survey.

### Sample size estimate and sampling technique

Taking into consideration the total number of live births in South West Cameroon for 2013 (12,861 births of approximately 6,687 urban and 6,174 rural), a 6 months data collection period, 95% confidence limit, and a 50% response distribution (worst case scenario), sample size needed was estimated at approximately 748 (374 for both urban and rural strata; estimated from openepi.com).

A two stage cluster sampling technique was used. In the first step, 20 out of 41 eligible hospitals (classified as rural or urban) were randomly selected. In the second step, all eligible women attending the selected hospitals during the study period were invited to participate.

### Data collection tools

A review of the literature was conducted to inform the initial design of the questionnaire (See S1 Questionnaire) for this study. The questionnaire had both open and closed ended questions, with measures to prevent recall bias (e.g. insisting that women identify their first three months of pregnancy, prior to completing the section on medication use). The questionnaire was pretested and validated prior to actual use. A picture guide of different herbs was developed to enhance women’s identification of the type of HM used.

### Recruitment and data collection

Eight nurse educators were trained as data collectors and provided with a guidance note to assist them during data collection. The pregnant women were recruited as they came for antenatal visits or, in a small number of cases, for hospital consultation. Women were first provided with verbal information about the study and later handed a copy of the study information sheet. Those who indicated interest to participate were requested to provide a written consent. Out of 817 eligible women approached, 795 agreed to participate in the study (97.3% response rate). Using the predesigned questionnaire and picture guide, the data collectors conducted one-on-one interviews for consented women in private rooms of the hospital to obtain data.

### Data analysis

Data were analysed using the descriptive and inferential statistical tools of the Statistical Package for the Social Sciences (SPSS) software, version 25.

We investigated hospital, women’s opinion on safety of OM or TM and safety advice. We also included the following commonly reported determinants of HM use: setting type (urban/rural), maternal age, marital status, education, living condition, gravidity, parity, gestational age at first ANC booking, pregnancy planning, and diseases during pregnancy. Differences in the prevalence of HM use within variables (all categorical) were tested using the Pearson Chi-squared test of independence. We then conducted a multivariate logistic regression analysis to test for independent contribution to HM use. A variable was included in the model if: (1) it was statistically significant in the univariate analysis, (2) it has strong support from the literature to be associated with HM use and (3) has a strong theatrical bases to influence HM use. In this report, we have presented only the results of variables that were included in both the univariate and multivariable model.

**Fig 1:**
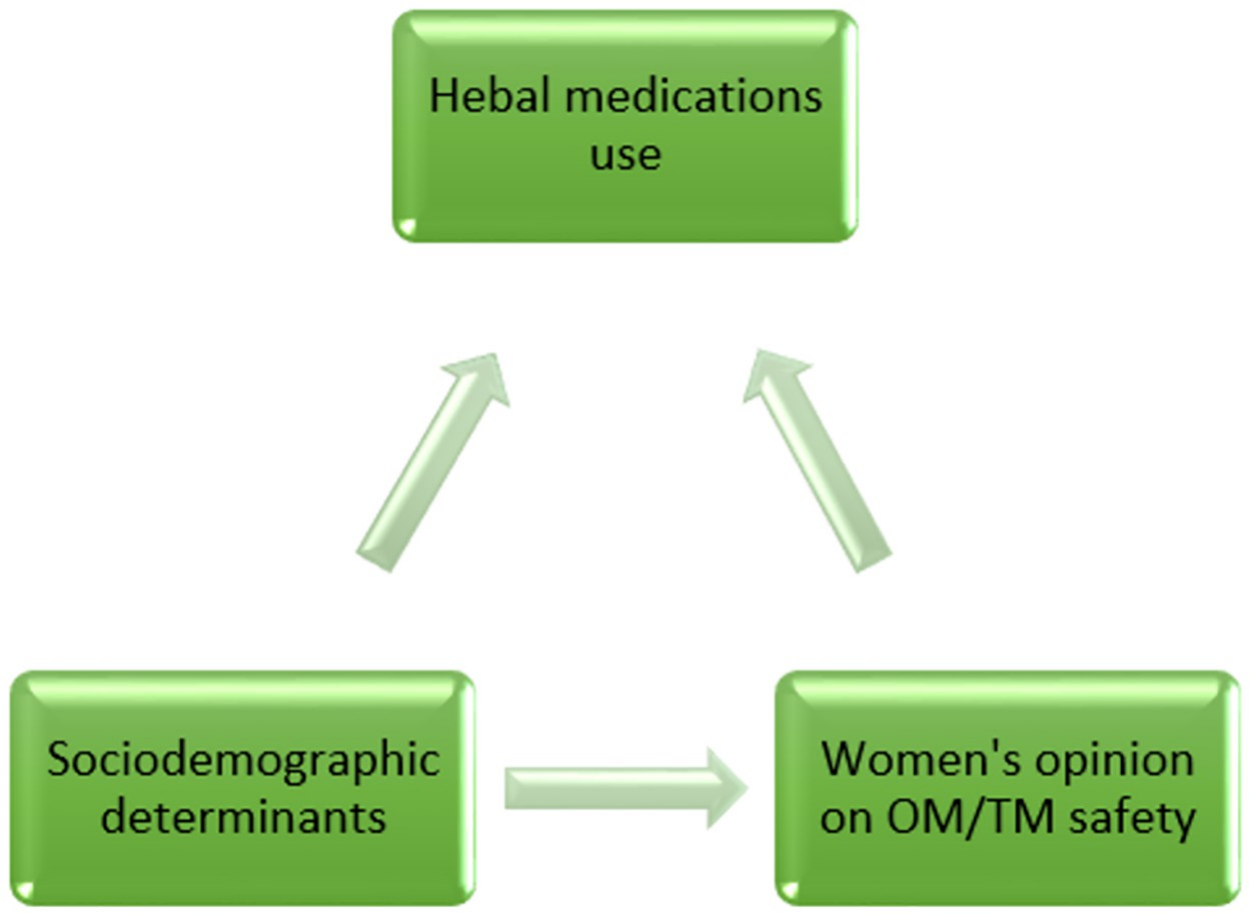
Relationship between sociodemographic factors, women’s OM/TM safety opinion and HM use.

There are two ways in which sociodemographic risk factors can affect HM use–either via an effect on the opinion of OM or TM safety, or independently of this opinion, or both (Fig 1). We therefore constructed a separate model to determine which sociodemographic determinants are associated with opinion of safety of OM or TM. Statistical significance level was set at 0∙05 and results were reported as adjusted odds ratios (AOR) and 95% confidence interval (95%CI).

### Ethical clearance

Ethical approval for this study was initially obtained from the Institute of Nursing and Health Research Governance Filter Committee of Ulster University and later from the National Ethics Committee in Cameroon (CNEC, Ref: 2014/08/489/CE/CNERSH/SP). The ethical review robustly covered all possible ethical concerns which were strictly observed throughout the study, including informed consent, confidentiality, data protection, privacy and right to withdraw from the study at any time during the interview.

## Results

Seven hundred and ninety-five women took part in the survey, 439 (55∙2%) from urban settings and 356 (44∙8%) from rural settings (Table 1).

**Table 1:** Proportion of women reporting HM use and adjusted odds ratios (by logistic regression) for determinants of HM use.

Determinants	Total (%)	At least one herbal medication (%)	Chi Squared P-value	Logistic regression model	
				OR (95%CI)	P-value¥
	**795(100)**	**293 (36.9)**			
**Hospital type**			**0.002**		**0.023**
Private^Ref^	174 (21.8)	84 (48.3)		-	
Government	621 (78.2)	209 (33.7)		**0.59 [0.38–0.93]**	
**Setting type**			**0.041**		0.198
Rural^Ref^	356 (44.8)	145 (40.7)		-	
Urban	439 (55.2)	148 (33.7)		1.27 [0.88–1.84]	
**Age (years)**			0.140		0.792
13-17^Ref^	41 (5.2)	19 (46.3)		-	
18–25	380 (47.8)	140 (36.8)		0.88 [0.40–1.94]	
26–35	335 (42.1)	119 (35.5)		0.92 [0.40–2.12]	
36–45	39 (4.9)	15 (38.5)		1.27 [0.41–3.91]	
**Marital status**			**0.032**		0.768
Married^Ref^	486 (61.1)	160 (32.9)		-	
Engaged	64 (8.1)	27 (42.2)		1.16 [0.61–2.22]	
Cohabitating (No formal engagement)	68 (8.6)	32 (47.1)		1.33 [0.71–2.49]	
Single	177 (22.3)	74 (41.8)		1.20 [0.76–1.89]	
**Level of Education**			0.109		0.580
Never went to school^Ref^	19 (2.4)	11 (57.9)		-	
Primary	205 (25.8)	77 (37.6)		0.61 [0.20–1.88]	
Secondary	329 (41.4)	119 (36.2)		0.62 [0.21–1.89]	
High School	129 (16.2)	53 (41.1)		0.61 [0.19–1.94]	
University/Professional	113 (14.2)	33 (29.2)		0.39 [0.11–1.37]	
**Living condition**			**<0.001**		**0.013**
House with Pit /external toilet^Ref^	597 (75.1)	232 (38.9)		-	
Renting self-contained studio	104 (13.1)	44 (42.3)		0.84 [0.48–1.48]	
Renting or Own a self-contained house	94 (11.8)	17 (18.1)		**0.35 [0.17–0.71]**	
**Number of diseases/ailments**			**<0.001**		**0.007**
0^Ref^	139 (17.5)	37 (26.6)		-	
1–3	410 (51.6)	138 (33.7)		**1.84 [1.10–3.09]**	
>3	246 (30.9)	118 (48.0)		**2.46 [1.41–4.31]**	
**Opinion on safety of Orthodox medications during pregnancy**			**<0.001**		**0.003**
Yes, it is always safe^Ref^	551 (69.3)	175 (31.8)		-	
Yes, safe but depends	157 (19.7)	75 (47.8)		**1.55 [1.00–2.41]**	
No, never safe	21 (2.6)	8 (38.1)		1.70 [0.57–5.07]	
I don’t know	66 (8.3)	35 (53.0)		**3.26 [1.68–6.34]**	
**Opinion on safety of traditional medication during pregnancy**			**<0.001**		**<0.001**
Yes, it is always safe^Ref^	162 (20.4)	109 (67.3)		-	
Yes, safe but depends	177 (22.3)	102 (57.6)		0.62 [0.38–1.01]	
No, never safe	319 (40.1)	45 (14.1)		**0.09 [0.05–0.15]**	
I don’t know	137 (17.2)	37 (27.0)		**0.16 [0.09–0.28]**	
**Participant received medication safety advice during current pregnancy**			0.846		0.377
Yes^Ref^	468 (58.9)	176 (37.6)		-	
No	287 (36.1)	102 (35.5)		1.28 [0.87–1.86]	
Can’t remember	40 (5.0)	15 (37.5)		0.93 [0.40–2.12]	

**Ref**: Reference category for logistic regression, **OR**: Odds ratio; **CI**: Confidence interval;

**^¥^**: Overal significance of variable within model

### Prevalence and factors associated with HM use

**Fig 2:**
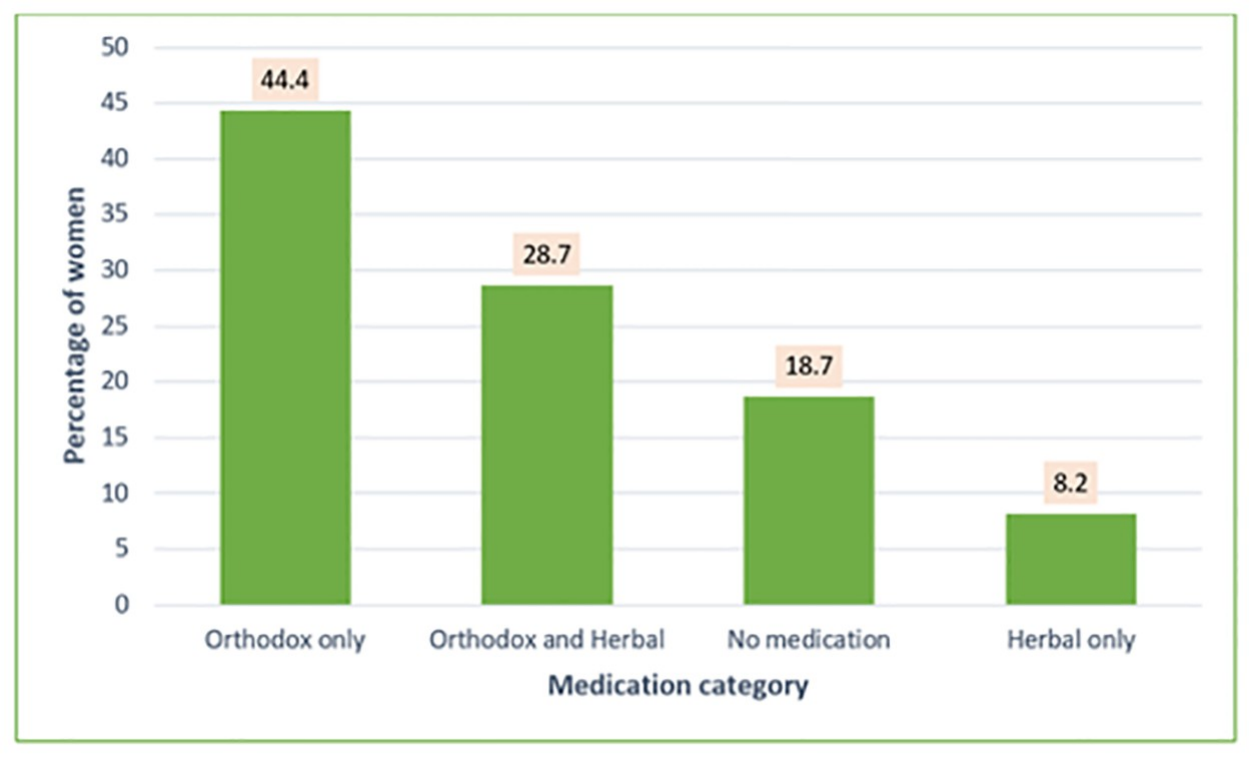
Overall first trimester use of orthodox and herbal medication (N = 795).

A total of 293 (36∙9%) women took at least one HM during the first trimester (Table 1), with 28∙7% concurrently using OM and HM (Fig 2). HM use was less common than OM use.

Consumption of HM was significantly higher among women attending private hospitals (48.3%), from rural settings (40∙7%), living in houses with pit/external toilets (38∙9%) or renting self-contained studio (42∙3%) (Table 1). A smaller proportion of married women (32∙9%) took HM. There was no association between HM use and maternal age or level of education (Table 1). Women who experienced more diseases or ailments were more likely to take HM (Table 1).

When asked about the safety of OM or TM, 69∙3% of women thought OM were always safe to use in pregnancy, compared 20∙4% of women who thought HM were always safe in pregnancy (Table 1). HM use was slightly higher among women who thought OM was never safe (38∙1%) compared to those who thought it was always safe (31.8%) (Table 1). Conversely, women who perceived TM as never safe were significantly less likely (14∙1%) to consume HM compared to those who thought TM was always safe (67.3%) (Table 1). Up to 58∙9% of women said they had received medication safety advice during the current pregnancy, but there was no association between HM use and receiving advice on medication safety during pregnancy (Table 1).

### Determinants of HM use

In the multivariate model of HM use, hospital type (p = 0∙023), living condition (p = 0∙013), number of diseases (p = 0∙007), opinion on safety of OM (p = 0∙016) and opinion on safety of TM (p < 0∙001) remained statistically significant factors explaining variation in HM use (Table 1). Women attending government hospitals had a 41% less chance of taking HM compared to those attending private hospitals. Women who were renting or owned a self-contained house were 65% less likely to take HM compared to those living in houses with pit/external toilet (AOR: 0.35, 95%CI = 0.17–0.71). The odds of using HM increased with number of diseases/ailments.

The 8.3% of women who were unsure of the safety of OM in pregnancy were particularly likely (AOR: 3∙0, 95%CI = 1∙5–6∙1) to take HM, while women who thought TM were never safe or who were unsure whether they were safe or not respectively had a 91% or 84% decrease in the odds of taking HM compared to women who believed TM were always safe. Women’s opinion on the safety of both OM and TM was associated with most of the sociodemographic variables studied (Appendix 1 and 2).

### Sources, prescribers, indications, and factors influencing decision to use HM

**Fig 3:**
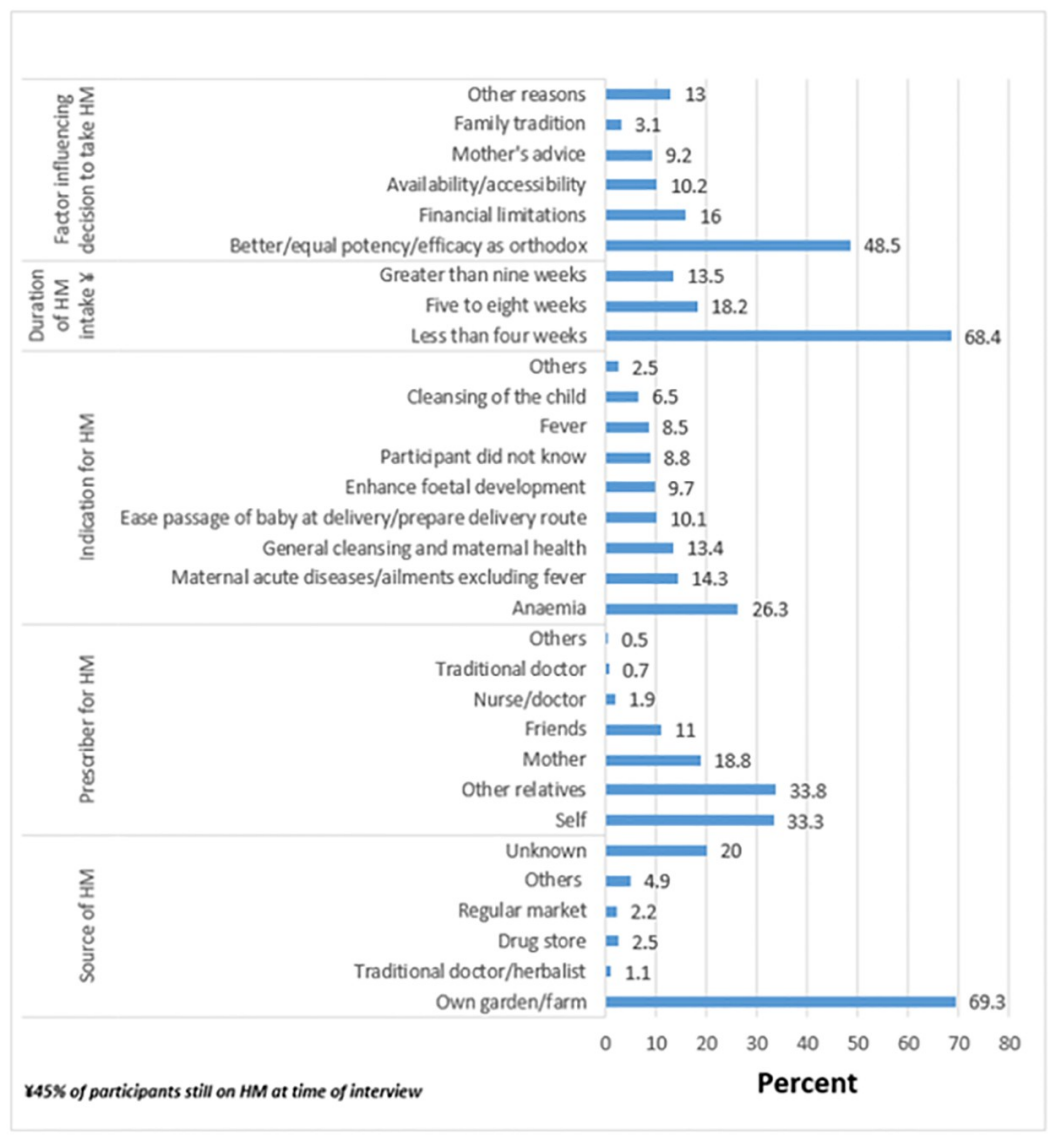
Sources, prescribers, indicators, duration of use and factors influencing use of HM.

The majority (69.3%) of women got their HM from their farms (Fig 3). HM were mainly self-prescribed (33.3%) or recommended by relatives (31.8%). The most frequent indication cited for HM use was to address the problem of anaemia (26.3%). However, a considerable proportion of women (8.8%) did not know why they were taking the HM. HM were taken for very long durations, with 45% of the participants still on the medications at the time of interview (Fig 3).

Women who took HM gave varied reasons on what guided their choice. Perceived better or equal potency of TM over OM was the most common factor cited (48.5% of women) influencing women’s decision to take HM (Fig 3).

## Discussion

### Prevalence and duration of first trimester HM use

First trimester HM use was common (36.9%) among women in our study. This prevalence is lower than reported in Nigeria (58.2%) [24] and Ethiopia (69.8%) [25], but higher than reported in Tanzania (6.8%) [26], Lesotho (30.9%) [27] and South Africa (2.7^%^) [28]. These differences of first trimester exposure to HM could be related to differences in African country contexts [15, 27–29] and/or study methodologies. For example, a common characteristic of the three studies reporting lower prevalence compared to our study was that they sampled women postpartum, with possible recall bias on first trimester HM use.

We identified a high proportion (28.7%) of women who took HM and OM concomitantly, increasing the possibility of interactions between the active substances with unknown safety and teratogenic potential [7, 15]. This is the first report of such a high proportion of concomitant use in Africa. Two reports in Ghana and Nigeria both estimated this at approximately 7% [30, 31]. This mixing of OM and HM adds to the existing problem of lack of identification of the active ingredients or medicinal properties of most African herbs, a major preoccupation of modern African phytotherapy/medicinal plants researchers [2, 6, 15]. Moreover, as reported elsewhere [15], women often use a mixture of herbs, and crude preparation methods, such as closing herbs in airtight bottles and allowing for a fermentation period before consumption.

Women in our study reported taking HM for very long durations across the first trimester, with 45% still using them at the time of interview. Indeed, 9% and 5% of women were constantly on HM throughout their pregnancy in the studies of Duru, Nnebue [32] in Nigeria and Bayisa, Tatiparthi [25] in Ethiopia, respectively. This long duration of intake, in combination with the lack of scientific dosage calculation, or knowledge on pharmacokinetics/pharmacodynamics emphasises the safety concern for pregnancy.

### Factors influencing decision to take HM

Corroborated by many other studies [15, 33, 34], most (48.5%) women who took HM in our study were influenced by the strong belief that HM were more effective compared to OM. Their perception could have been shaped by cultural beliefs; failure of OM to treat resistant infections such as malaria and emerging conditions such as HIV/AIDS, cancer, diabetes and hypertension; and/or poverty [2, 6, 7]. Other studies have reported the fact that herbs are ‘natural’ and safer as common reasons for women’s preference of HM over OM [20, 36]. This is also a central argument put forward by proponents of HM [2, 31]. However, very few women in our study reported these factors as positive reasons for their choice, although their opinion on safety did affect their willingness to take HM, perhaps via non-avoidance.

### Source of HM

To address the problems of lack of identification of active ingredients, dosage and pharmacokinetics/pharmacodynamics, modern proponents of HM in Africa are beginning to design packaged forms of herbs following scientific procedures [1, 6]. This form of HM is what is widely available in the Western world today. However, most (69.3%) of the women who used herbs in our study obtained them from their farms/forest, with less than 3% reporting use of HM from drug store which are usually available in the packaged form. This observation is true for many other studies in Africa [15]. A few studies have cited traditional herbalists as a common source [30, 36], but very few (about 1%) women in our study reported this source. Women’s preference to obtain herbs from their farms may be associated with availability, accessibility and low cost [14].

### Prescribers and indications of HM

One third of the women in our study self-prescribed HM, but their mothers and other relatives were also major prescribers. These results are similar to those reported by many other studies in Africa [30, 34–36], and are possibly a reflection of the shift in access to HM knowledge. In the past, the knowledge and practice of HM in Africa was reserved for a ‘chosen few’ in the community (herbalists, traditional doctor, spiritualist) [6, 7]. It is believed that they received this knowledge from supernatural beings who either abducted them into the spiritual realm where they were trained, or provide such training via revelations/vision/dreams [7]. In modern times however, knowledge of HM is widespread among ‘ordinary’ people in the population. Specialised training in HM is even now possible in some academic institutions (e.g. in Ghana and South Africa) [37]. Self-prescription of HM increases the woman’s risk of exposure to teratogenic herbs. It also delays her from seeking medical care, potentially leading to the delay of important diagnosis such as preeclampsia and gestational diabetes.

Consistent with other studies, women in our study took HM in the first trimester to address different problems which can be summarised into two main categories: to enhance maternal/fetal wellbeing and ease delivery (39.7%), and to prevent or treat diseases or ailments (49.1%) [15, 32, 34, 36–39]. However, it is important to note that the types of HM taken and their indication depend on the period of pregnancy. For example, during the perinatal period, women choose HM which have ecbolic effects [40–42].

Anaemia was the most reported indication for HM use, as reported by many other studies in Africa [16, 32, 40, 43]. The prevalence of anaemia in our study population had been previously estimated at 70% [44]. Prevention of anaemia is therefore central to the antenatal care programme which almost all women in our study attended. Malaria is an important contributor to anaemia during pregnancy in Cameroon. During visits, women are given (free of charge) sulfadoxine pyrimethamine (SP) intermittently for the prevention of malaria, as well as prescribed blood supplements. The administration of SP during antenatal clinics has several challenges causing uptake by pregnant women to be very low in most African countries [45]; e.g. medication not always available, high workload of nurses, debates about its effectiveness, and lack of awareness among pregnant women and nurses. Another major challenge is the high cost and growing resistance to orthodox antimalarial medications meant for treatment [46]. These challenges may be viewed as contributing to pregnant women choosing to use HM.

### Determinants of HM use

After accounting for the presence of other variables in multivariate analysis, the main factors significantly contributing to HM use were the type of hospital attended, living condition, number of diseases/ailments, and opinion on the safety of OM or TM.

HM are rarely prescribed in the hospital, but surprisingly, even after accounting for women’s individual characteristics and their opinion on safety, type of hospital persisted as a strong determinant of HM use—women attending private hospitals were significantly more likely to take HM compared to those attending government hospitals. This relationship between type of hospital and HM intake could be attributed in part to the attitude of healthcare practitioners toward intake of TM. Evidence suggests some nurses and midwives have negative attitudes towards women who take TM [20, 37], while some hospital policies may create a more permissive attitude [17], particularly in private hospitals where the cost of care is higher, but patients have more rights and freedom to choose their care options. Indeed, many studies have reported that the majority of patients fail to disclose HM use to their healthcare provider [4, 20, 37]. On the other hand, healthcare providers frequently fail to question women about HM use [17].

To the best of our knowledge, this is the first report to examine women’s opinion on TM/OM safety as a determinant of HM use. Women’s opinion on the safety of OM or TM was strongly associated with sociodemographic determents of HM use. We therefore hypothesise that sociodemographic risk factors influence HM use via two routes–via an effect on the opinion of OM or TM safety, and independently of this opinion. The important role of perception of OM or TM safety should therefore be factored in when planning interventions for HM safety in pregnancy. This need is further emphasised by data from our study indicating a third of the women had not received any medication safety advice.

Many studies have found that women with low level of education are significantly more likely to take HM [4, 24, 32, 36, 38]. Although a higher proportion of women with low level of education in our study took HM, this difference was not large or statistically significant. However, we found that level of education was significantly associated with women’s opinion on OM and HM safety, which was in turn associated with HM use.

### Pregnancy safety considerations for integrating TM into healthcare systems in Africa

According to the 2019 report of the WHO Global Programme on Traditional, Complementary and Integrative Medicine, national policies, laws/regulations and programmes have been derived on traditional and complementary medications (T & CM) in most WHO Member states in Africa [10]. However, dealing with the challenges on how to integrate T & CM into the existing healthcare systems remains an uphill challenge for many nations [10, 22, 47]. The WHO initiative to integrate T & CM into the existing healthcare systems could enhance access to much needed treatment in Africa. However, this initiative must recognise the fact that pharmacovigilance systems in Africa are still very weak and should pay special attention to the safety of both OM and TM in vulnerable groups such as pregnant women.

There is very limited or no evidence on the safety of HM used during pregnancy, particularly in Africa. A recent systematic global review examining studies on adverse effects of HM during pregnancy found 74 studies, with only 7 examining data on first trimester use and 19 investigating congenital anomalies as a primary outcome [48]. Furthermore, only 47 medicinal products (single substance or a mixture of plants) were examined. The majority of these studies were undertaken in western countries and therefore examined only a few plant products that are found or commonly used in the African context. Only one unique African herbal preparation—Mwanaphepo, used for labor induction, was investigated for adverse effects and found to be associated with several maternal complications [49]. This means there is still no pregnancy safety data for the over 274 plant species that have been reported to be used by pregnant women across Africa [15].

Furthermore, information about herbal medicines should be included in birth defect prevention programs. Currently, the Global Birth Defects inventory of resources has begun to list existing resources [50], but there is a need for the development of further resources for health professionals and mothers and to strengthen pharmacovigilance systems to monitor the safety of HM in pregnancy in addition to OM.

### Strengths and limitations

In contrast to other studies conducted in Africa [13], our study on HM use focused exclusively on the first trimester of pregnancy, the period of highest teratogenic risk. We employed a robust approach training research nurses as data collectors, targeting only women within 3–7 months gestation, and using a picture guide for identification of herbal medicines to enhance women’s reporting of HM intake and limit recall bias. We included a large sample of hospitals and women, and modelled for the first time, the influence women’s opinion on TM/OM safety on HM use.

Unfortunately, due to limited resources it was not possible in this study to verify the scientific names of the herbs used. While we collected data on local names, as herbs have different local names within countries, regions and tribes, without their scientific names, it is impossible to make full use of this information, or to make comparisons across countries and regions. Although our interviews were conducted in private, away from the healthcare personnel, we cannot exclude the possibility that knowledge of hospital policy influenced women’s readiness to disclose HM use or positive opinions about its safety. Notwithstanding, the data suggests a strong influence of healthcare provider on HM-related behaviour which needs further research.

## Conclusions

Our study identified a high use of HM during the first trimester of pregnancy, as well as concomitant use of HM and OM, raising safety concerns. We found that use of HM was strongly influenced by women’s perception of the safety of both OM and TM and varied considerably between government and private hospitals. Coming at a time when Africa, led by WHO, is advocating for the reintegration of TM practice into existing healthcare systems, these results should help put medication safety in pregnancy within this agenda.

## Supporting information

S1 Table Factors associated with maternal opinion on safety of orthodox medications during pregnancy. (DOCX)

S2 Table Factors associated with maternal opinion on safety of traditional medications during pregnancy. (DOCX)

S1 Questionnaire Questionnaire-based interview. (DOCX)
