# Supplementary material for: Prevalence, determinants and attitude towards herbal medicine use in the first trimester of pregnancy in Cameroon: A survey in 20 hospitals
Source: PLOS Glob Public Health. 2022 Aug 30;2(8):e0000726. doi: 10.1371/journal.pgph.0000726 (PMC10021538; doi:10.1371/journal.pgph.0000726)
Supplement: S1 Table — (DOCX) [file pgph.0000726.s001.docx]

| Factor | Opinion on safety of orthodox medication during pregnancy | | | | Chi Sq P-value |
| --- | --- | --- | --- | --- | --- |
|  | **Yes, it is always safe**  **n (%)** | **Yes, safe but depends**  **n (%)** | **No, Never safe**  **n (%)** | **I don't Know**  **n (%)** |  |
| Setting type |  |  |  |  | 0.143 |
| Urban | 234(65.7) | 74(20.8) | 11(3.1) | 37 (10.4) |  |
| Rural | 317(72.2) | 83(18.9) | 10(2.3) | 29(6.6) |  |
| Age (years) |  |  |  |  | 0.005 |
| 13-17 | 262(68.9) | 71(18.7) | 11(2.9) | 36(9.5) |  |
| 18-25 | 23(56.1) | 6(14.6) | 4(9.8) | 8(19.5) |  |
| 26-35 | 237(70.7) | 74(22.1) | 6(1.8) | 18(5.4) |  |
| 36-45 | 29(74.4) | 6(15.4) | 0(0.0) | 4(10.3) |  |
| Marital status |  |  |  |  | 0.050 |
| Married | 352(72.4) | 92(18.9) | 9(1.9) | 33(6.8) |  |
| Divorced | 5(83.3) | 1(16.7) | 0(0.0) | 0(0.0) |  |
| Engaged | 47(73.4) | 12(18.8) | 0(0.0) | 5(7.8) |  |
| Cohabitating (No formal engagement) | 47(69.1) | 11(16.2) | 2(2.9) | 8(11.8) |  |
| Single | 100(58.5) | 41(24.0) | 10(5.8) | 20(11.7) |  |
| Level of Education |  |  |  |  | 0.002 |
| Never went to school | 238(72.3) | 52(15.8) | 10(3.0) | 29(8.8) |  |
| Primary | 12(63.2) | 3(15.8) | 0(0.0) | 4(21.1) |  |
| Secondary | 149(72.7) | 34(16.6) | 3(1.5) | 19(9.3) |  |
| High School | 86(66.7) | 29(22.5) | 3(2.3) | 11(8.5) |  |
| University/Professional | 66(58.4) | 39(34.5) | 5(4.4) | 3(2.7 ) |  |
| Living condition |  |  |  |  | <0.001 |
| House with Pit /external toilet | 432(72.4) | 97(16.2) | 13(2.2) | 55(9.2) |  |
| Renting self-contained studio | 66(63.5) | 28(26.9) | 3(2.9) | 7(6.7) |  |
| Renting or Own a self-contained house | 53(56.4) | 32()34.0 | 5(5.3) | 4(4.3) |  |
| Number of diseases/ailments |  |  |  |  | <0.001 |
| 0 | 90(64.7) | 20(14.4) | 3(2.2) | 26(18.7) |  |
| 1-3 | 308(75.1) | 65(15.9) | 11(2.7) | 26(6.3) |  |
| >3 | 153(62.2) | 72(29.3) | 7(2.8) | 14(5.7) |  |
| Participant receives medication safety advice during current pregnancy |  |  |  |  | <0.001 |
| Yes | 337 (72.0) | 100(21.4) | 10(2.1) | 21(4.5) |  |
| No | 193(67.2) | 52(18.1) | 9(3.1) | 33(11.5) |  |
| Can't remember | 21(52.5) | 5 (12.5) | 2(5.0) | 12(30.0) |  |
