# Supplementary material for: Prevalence, determinants and attitude towards herbal medicine use in the first trimester of pregnancy in Cameroon: A survey in 20 hospitals
Source: PLOS Glob Public Health. 2022 Aug 30;2(8):e0000726. doi: 10.1371/journal.pgph.0000726 (PMC10021538; doi:10.1371/journal.pgph.0000726)
Supplement: S2 Table — (DOCX) [file pgph.0000726.s002.docx]

| Factor | Opinion on safety of traditional medication during pregnancy | | | | Chi Sq  P-value |
| --- | --- | --- | --- | --- | --- |
|  | **Yes, it is always safe**  **n (%)** | **Yes, safe but**  **depends**  **n (%)** | **No, Never safe**  **n (%)** | **I don't Know**  **n (%)** |  |
| Setting type |  |  |  |  | <0.001 |
| Urban | 70 (15.9) | 84 (19.1) | 207 (47.2) | 78 (17.8) |  |
| Rural | 92 (25.8) | 93 (26.1) | 112 (31.5) | 59 (16.6) |  |
| Age (years) |  |  |  |  | 0.026 |
| 13-17 | 15 (36.6) | 4 (9.8) | 13 (31.7) | 9 (22.0) |  |
| 18-25 | 69 (18.2) | 92 (24.2) | 146 (38.4) | 73 (19.2) |  |
| 26-35 | 73 (21.8) | 72 (21.5) | 145 (43.3) | 45 (13.4) |  |
| 36-45 | 5 (12.8) | 9 (23.1) | 15 (38.5) | 10 (25.6) |  |
| Marital status |  |  |  |  | 0.534 |
| Married | 92(18.9) | 100(20.6) | 209(43.0) | 85(17.5) |  |
| Divorced | 2(33.3) | 1(16.7) | 2(33.3) | 1(16.7) |  |
| Engaged | 15(23.4) | 19(29.7) | 20(31.3) | 10(15.6) |  |
| Cohabitating (No formal engagement) | 18(26.5) | 13(19.1) | 22(32.4) | 15(22.1) |  |
| Single | 35(20.5) | 44(25.7) | 66(38.6) | 26(15.2) |  |
| Level of Education |  |  |  |  | 0.002 |
| Never went to school | 6 (31.6) | 6 (31.6) | 3 (15.8) | 4 (21.1) |  |
| Primary | 59 (28.8) | 30 (14.6) | 81 (39.5) | 35 (17.1) |  |
| Secondary | 56 (17.0) | 75 (22.8) | 142 (43.2) | 56 (17.0) |  |
| High School | 27 (20.9) | 36 (27.9) | 40 (31.0) | 26 (20.2) |  |
| University/Professional | 14 (12.4) | 30 (26.5) | 53 (46.9) | 16 (14.2) |  |
| Living condition |  |  |  |  | 0.009 |
| House with Pit /external toilet | 130 (21.8) | 129 (21.6) | 236 (39.5) | 102 (17.1) |  |
| Renting self-contained studio | 21 (20.2) | 33 (31.7) | 32 (30.8) | 18 (17.3) |  |
| Renting or Own a self-contained house | 11 (11.7) | 15 (16.0) | 51 (54.3) | 17 (18.1) |  |
| Number of diseases/ailments |  |  |  |  | <0.001 |
| 0 | 29 (20.9) | 16 (11.5) | 59 (42.4) | 35 (25.2) |  |
| 1-3 | 72 (17.6) | 86 (21.0) | 181 (44.1) | 71 (17.3) |  |
| >3 | 61 (24.8) | 75 (30.5) | 79 (32.1) | 31 (12.6) |  |
| Participant receives medication safety advice during current pregnancy |  |  |  |  | <0.001 |
| Yes | 77 (16.5) | 122 (26.1) | 207 (44.2) | 62 (13.2) |  |
| No | 77 (26.8) | 49 (17.1) | 101 (35.2) | 60 (20.9) |  |
| Can't remember | 8 (20.0) | 6 (15.0) | 11 (27.5) | 15 (37.5) |  |
